# Biochemical and *in silico* characterization of glycosyltransferases from red sweet cherry (*Prunus avium* L.) reveals their broad specificity toward phenolic substrates

**DOI:** 10.1016/j.fochms.2023.100193

**Published:** 2023-12-31

**Authors:** Daniel Clayton-Cuch, Long Yu, Daniel McDougal, Crista A. Burbidge, John B. Bruning, David Bradley, Christine Böttcher, Vincent Bulone

**Affiliations:** a Adelaide Glycomics, University of Adelaide, School of Agriculture, Food and Wine, Waite Campus, Adelaide, South Australia 5064, Australia; b CSIRO, Waite Campus, Glen Osmond, South Australia 5064, Australia; c Institute for Photonics and Advanced Sensing (IPAS), School of Biological Sciences, The University of Adelaide, Adelaide, South Australia 5005, Australia; d Agilent Technologies Australia Pty Ltd, Mulgrave, Melbourne, Victoria 3171, Australia; e Division of Glycoscience, Department of Chemistry, School of Engineering Sciences in Chemistry, Biotechnology and Health, Royal Institute of Technology (KTH), AlbaNova University Centre, Stockholm 10691, Sweden

**Keywords:** Anthocyanins, Cherry, Glycosyltransferase, Flavonols, Protein structure modelling, Phenolic compounds

## Abstract

Polyphenolic compounds are a class of phytonutrients that play important roles in plants and contribute to human health when incorporated into our diet through fruit consumption. A large proportion occur as glycoconjugates but the enzymes responsible for their glycosylation are poorly characterized. Here, we report the biochemical and structural characterization of two glycosyltransferases from sweet cherry named *Pa*UGT1 and *Pa*UGT2. Both are promiscuous glucosyltransferases active on diverse anthocyanidins and flavonols, as well as phenolic acids in the case of *Pa*UGT1. They also exhibit weaker galactosyltransferase activity. The expression of the gene encoding *Pa*UGT1, the most active of the two proteins, follows anthocyanin accumulation during fruit ripening, suggesting that this enzyme is the primary glycosyltransferase involved in flavonoid glycosylation in sweet cherry. It can potentially be used to synthesize diverse glycoconjugates of flavonoids for integration into bioactive formulations, and for generating new fruit cultivars with enhanced health-promoting properties using breeding methods.

## Highlights

• Two glycosyltransferases from sweet cherry, *Pa*UGT1 and *Pa*UGT2, are described.
• Both show glucosyltransferase and weaker galactosyltransferase activities.
• Both are active on diverse anthocyanidins, flavonols and phenolic acids.
• *Pa*UGT1 is a key enzyme for flavonoid glycosylation in sweet cherry.
• *Pa*UGT1 adopts glycosyltransferase B fold and uses conventional transfer mechanism.

## 1 Introduction

Phytonutrients are a large family of secondary metabolites present in varying concentrations and combinations in all commonly consumed crops worldwide, including fruits and vegetables. They play various important roles within the plant itself and promote human health by contributing to the prevention of diseases (Zhang et al., 2022). Among the significant subclasses of phytonutrients, phenolic compounds exhibit potent anti-inflammatory and anti-carcinogenic activities when consumed (Zhang et al., 2022). This subclass alone comprises more than 8,000 structures, including phenolic acids, flavonoids, stilbenes, lignans, tannins and proanthocyanidins (Zhang et al., 2022). Anthocyanins, flavonols and phenolic acids in particular have been widely studied, with a plethora of literature reporting on their biosynthesis, biological activities, abundance in various sources, and potential for enhancing bioactivities (Kumar & Goel, 2019).

Due to the many health benefits associated with consumption of phenolic compounds, there is great interest in manipulating their biosynthesis to further enhance the nutritional value of fruit and vegetables. While substantial research has been conducted on the biosynthesis of these potent bioactives, certain steps, such as glycosylation, remain poorly understood. Glycosylation masks hydroxyl groups of phenolic compounds, protecting them from oxidation and conferring structural stability, which allows for their presence in the diverse plant tissues (Xu et al., 2016). Flavonoids and phenolic acids are found in both aglycone and glycoside forms in plants. However, glycoside forms are far more abundant than their aglycone counterparts (Hollman, 2004).

These ubiquitous phenolic compounds encompass a diverse range of structures. Flavonoids consist of a C6-C3-C6 ring structure where two aromatic rings, A and B, are connected by three carbon atoms, which may lead to the formation of a third C ring. On the other hand, phenolic acids display a C6-C3 structure characterized by a phenolic ring and a carboxylic acid group. These compounds serve as precursors in the biosynthesis of lignans and other phenolics (Kumar & Goel, 2019). Variations within this basic structure give the various subclasses of phenolic compounds. Flavonoids are typically glycosylated at the O3 position in ring C or O7 position in ring A. Although less commonly reported, glycosylation at the O5 position in ring A has also been observed (Hofer, 2016). The sugars that are most typically attached to these compounds include glucose or galactose, but other sugars such as xylose, arabinose, and glucuronic acid have also been widely reported (Zhang et al., 2020). These diverse glycosylation patterns contribute to the properties of the different phenolic compounds.

Interestingly, although the glycosylation events in the biosynthesis of many phenolic compounds have not been precisely characterized, there is a wealth of enzymes that have been identified as putatively involved in catalysing these reactions. In some species, such as apple (*Malus domestica* Borkh.), previous studies have described the substrate specificity of putative UDP glycosyltransferase enzymes (UGT). For example, *Md*UGT75B1 and *Md*UGT71B1 were shown to act on major flavonols in apple, including quercetin, phloretin, kaempferol and naringenin (Xie et al., 2020). Another study identified *Md*UGT83L3 as an apple UGT enzyme capable of glycosylating both anthocyanidins and flavonols using UDP-glucose as a sugar donor (Li et al., 2022). More recently *Md*UGT78T2 was identified amongst 234 other putative apple glycosyltransferases and shown to have dual activity on anthocyanidins and flavonols, forming the major phenolic compounds in red apple, namely quercetin-3-*O*-galactoside and cyanidin-3-*O*-galactoside, respectively (Clayton-Cuch et al., 2023). These studies exemplify the significant progress made towards the complete characterization of the glycosylation events involved in the biosynthesis of the primary phenolic compounds found within this important crop.

However, knowledge gaps persist, particularly in the case of cherry (*Prunus avium* L.), a globally consumed fruit with abundant phytonutrients that play an important role in human health and disease prevention (Gonçalves et al., 2022). Although several putative UGTs have been identified in cherry, mainly through transcriptomics or genomic approaches, their actual biochemical functions have not been experimentally demonstrated (Clayton-Cuch et al., 2021, Jin et al., 2016, Liang et al., 2020, Shen et al., 2014, Starkevič et al., 2015, Wang et al., 2023). Consequently, there is no evidence of how broadly these putative UGTs act on the diverse classes of phenolic compounds found within cherry. One noteworthy UGT identified in previous studies as a key glycosyltransferase in cherry is *Pa*UGT1. Substantial evidence indicates that the expression profile of this enzyme correlates with the accumulation pattern of anthocyanins in cherry (Clayton-Cuch et al., 2021, Jin et al., 2016, Liang et al., 2020, Shen et al., 2014, Starkevič et al., 2015, Wang et al., 2023). The present work focused on characterizing this UGT, with particular emphasis on identifying the diversity of sugar acceptors it can utilize as substrates, as well as the specific nucleotide-sugar donors it commonly employs. RNA-Seq data revealed that *PaUGT2*, another UGT-encoding gene similar to *PaUGT1*, is expressed throughout cherry development (Wei et al., 2015). The biochemical characterization of the corresponding enzyme was also undertaken in our study. By gaining a deeper understanding of more cherry UGTs and their substrate specificity, new breeding approaches may emerge to enhance the nutritional value of fruits and their health benefits.

## 2 Materials and methods

### 2.1 Chemicals

Promega’s ‘Ultra-pure’ UDP-galactose (UDP-Gal) and UDP-glucose (UDP-Glc) were used as sugar donors in UGT assays (Madison, WI, USA). Cyanidin chloride, peonidin chloride, malvidin chloride, quercetin, kaempferol, isorhamnetin, naringenin, catechin, epicatechin, caffeic acid, chlorogenic acid, quinic acid, coumaric acid and ferulic acid standards were purchased from Sigma Aldrich (St. Louis, MO, USA). All standards were of HPLC grade and diluted to 100 mM in DMSO and stored at –20 °C until use. Acetonitrile, methanol, and formic acid (LC-MS grade) were purchased from Fisher Scientific (Waltham, MA, USA).

### 2.2 Fruit material

In 2020, mature Lapins (*Prunus avium* L.) trees grafted on Colt rootstocks in a commercial orchard at Lenswood, South Australia (lat. 34°54′04.8″S; long. 138°48′54.2″E) were used to sample developing cherries. Three replicates of 24 cherries each were randomly collected from two trees per replicate at 23, 29, 37, 44, 51, 58, 72, 79 and 86 days after flowering (i.e., days after full bloom; DAF), weighed (PB3002-S; Mettler-Toledo, Melbourne, Australia), and immediately frozen in liquid nitrogen and stored at –60 °C. After removal of pedicels and pits, the frozen pericarp tissues were ground to a powder using an IKA A11 basic analytical mill (IKA, Staufen, Germany), stored at –80 °C and used for RNA extractions (see below). Subsamples (500 mg) of the cherry powder were thawed and used for the measurement of total soluble solids using an RFM710 digital refractometer (Bellingham Stanley, Kent, UK).

### 2.3 *In silico* screening for cherry UGTs involved in the anthocyanin biosynthetic pathway

Publicly available RNA-seq data (Wei et al., 2015) of four different developmental stages (20 DAF, 35 DAF, 45 DAF and 55 DAF) of the pure yellow cherry cultivar 13-33 and the red cultivar Tieton were filtered to identify sequences containing the PFAM PF00201 motif, which is a signature sequence of glycosyltransferase enzymes from family 1 (GT1) including UDP-Glc, UDP-Gal and UDP-glucuronosyl (UDP-GlcA) transferases (Fan et al., 2017; Finn et al., 2014). Further filtering was performed on sequences containing the PFAM PF00201 motif to detect those with increased expression levels in the red compared with the yellow cultivar.

### 2.4 RNA extraction, cDNA synthesis and qRT-PCR

Total RNA was extracted from the tissue homogenates using the Sigma Spectrum Plant Total RNA Kit (Sigma-Aldrich, MO, USA). RNA integrity was tested by agarose gel electrophoresis and RNA concentration and quality were determined spectrophotometrically using a Thermo Scientific NanoDrop microvolume instrument (Thermo Fisher Scientific, MA, USA). Following RNA extraction, samples were incubated in the presence of DNase I (New England Biolabs, MA, USA) following the manufacturer’s instructions, and RNA was quantified again as described above. DNase treated RNA (1 µg) was reverse transcribed using the Invitrogen Superscript IV RT enzyme (Invitrogen, CA, USA), according to the manufacturer’s instructions. The synthesized cDNAs were diluted 1:20 in RNase/DNase-free water. Fragments amplified using gene-specific primers designed from the 3′-untranslated regions (Supplementary Table S1) were purified by HPLC (Clayton-Cuch et al., 2021), and sequenced at the Australian Genome Research Facility (Adelaide, South Australia). qRT-PCR analyses were performed using the Roche LightCycler 480 System as described previously (Böttcher et al., 2015) and a Qubit 2.0 Fluorometer (Invitrogen, CA, USA) to quantify the DNA in the purified amplicon standards. Transcript levels of the gene encoding elongation factor 1-alpha (EFA) were used as a reference (Supplementary Table S1).

### 2.5 Extraction and analysis of phenolic compounds

Frozen and ground pericarp tissues from cherry samples collected at 72 DAF were freeze-dried to complete dryness (Dynavac freeze dryer Model FD-1C-50, MA, USA). The samples (∼50 mg) were then mixed with 70 % methanol, stirred at 25 °C for 1 h, and centrifuged at 10,000 *g* for 20 min. The supernatants were diluted 1:9 with 50 % methanol for liquid chromatography and mass spectrometry (LC-MS) analysis of phenolic compounds. For this purpose, an Agilent ultra-high performance LC instrument coupled to an Agilent 6540 Quadrupole Time-of-Flight (QTOF) mass spectrometer (Agilent Technologies, CA, USA) was used. The LC system was fitted with an Agilent Zorbax Eclipse C18 column (100 mm × 2.1 mm, 1.8 µm) and the column temperature was maintained at 40 °C. Five µL of samples was injected and phenolic compounds were separated at a flow rate of 0.3 mL/min using a gradient of eluent A (0.1 % formic acid in H_2_O) and eluent B (90 % acetonitrile in 0.1 % formic acid) as follows: 2 % B (0–1 min), 2 %–30 % B (1–16 min), 30 %–100 % (16–18 min), 100 % B (18–19 min), 100 %–2% B (19–21 min). After elution, the column was re-equilibrated for 5 min in 2 % B. A diode-array detector (DAD) connected to the LC-MS instrument was set to record spectra from 270 to 550 nm. The source parameters of the mass spectrometer were as follows: ESI interface; negative or positive mode; auto MS/MS scan; nebulizer, 35 psi; gas temperature, 220 °C; gas flow rate, 18 L/min; sheath gas temperature, 300 °C; sheath gas flow rate, 12 L/min; capillary voltage, 3000 V; MS range from *m*/*z* 100 to 1500; collision energy, 20 V. All data were processed using the MassHunter qualitative analysis software version 10.0 (Agilent Technologies, CA, USA). Compounds were identified based on their observed *m*/*z* values and MS/MS product ions as well as by comparison with data available in the literature (Martini et al., 2017).

### 2.6 Expression of recombinant UGT proteins in *Escherichia coli* and purification

The full-length open reading frame sequences of the cherry UGT genes *PaUGT1* and *PaUGT2* (Supplementary Table S2) were individually synthesized into a pET-30b (+) plasmid by GenScript Biotech (Piscataway, NJ, USA) to generate *Pa*UGT1/2-His as C-terminal fusion proteins. *Escherichia coli* BL21 (DE3) Origami 2 cells (Merck, Darmstadt, Germany) were co-transformed with the pRIL plasmid (Stratagene, La Jolla, CA, USA) and the recombinant pET-30b (+) plasmid containing either *PaUGT1* or *PaUGT2***.**

Cells were grown at 37 °C until an OD (600 nm) of 0.6–0.8 was reached. They were subsequently placed on ice for 10 min, after which protein expression was induced with 0.2 mM isopropyl-β-D-thiogalactopyranoside. The cultures were incubated for 24 h at 18 °C in an orbital shaker set at 170 rpm, and the cells were harvested by centrifugation at 10,000 × g for 15 min (4 °C), frozen in liquid nitrogen and thawed on ice for 20 min. Following three consecutive freeze–thaw cycles, the cells were resuspended in 20 mM sodium phosphate buffer pH 7.4 containing 500 mM sodium chloride, 20 mM imidazole and one tablet of EDTA-free protease inhibitor cocktail per 10 mL buffer (Merck, Darmstadt, Germany). The cells were disrupted by sonication for 15 min and the samples were centrifuged at 35,000 × g for 20 min at 4 °C. The recombinant UGT proteins present in the supernatants were purified using a His GraviTrap Ni Sepharose^TM^ 6 Fast Flow column (GE Healthcare, Buckinghamshire, UK). The molecular mass of the purified recombinant proteins was verified by SDS-PAGE using 4–20 % Mini-PROTEAN TGX acrylamide gels (Bio-Rad, CA, USA). The purified proteins were concentrated using an Amicon Ultra-4 Centrifugal 50 K Filter Unit (Merck, Darmstadt, Germany) and protein concentration was measured by spectrophotometry using a Thermo Scientific NanoDrop microvolume instrument (Thermo Fisher Scientific, MA, USA).

### 2.7 UGT assay and LC-MS/QQQ analysis of UGT reaction products

The activity of the purified recombinant *Pa*UGT1 and *Pa*UGT2 proteins was measured in 96-well plates using the UDP-Glo^TM^ Glycosyltransferase Assay Kit, which involves a modified luciferase for the detection of the free UDP released during the glycosyltransferase reaction (Promega, WI, USA). A range of sugar acceptor substrates were tested in the presence of the donors UDP-Glc or UDP-Gal to determine the specificity of *Pa*UGT1 and *Pa*UGT2. Reactions were carried out for 1 h at 30 °C in 50 µL reaction mixtures containing 50 mM Tris-HCl pH 8.0, 1 mM sugar donor, 500 µM sugar acceptor and 10 µg protein. Following catalysis, one volume of the UDP Detection Reagent provided in the kit was added to the mixtures to concomitantly stop the reactions, convert the free UDP to ATP, and generate light in the presence of luciferase. Luminescence was measured using a FLUOstar Omega filter-based multi-mode microplate reader (BMG LABTECH, Ortenberg, Germany) and the amount of light emitted was correlated to UDP concentration using a UDP standard curve. All substrates were diluted from DMSO stock solutions stored at –20 °C and all reactions were performed in triplicate.

To confirm the formation of glycoconjugates and their identity, and to demonstrate that the *Pa*UGT1 and *Pa*UGT2 reactions were catalytic, reaction mixtures were prepared as indicated above in the presence of either 1, 10 or 20 µg of each recombinant protein, and the products formed after 1 h at 30 °C were analyzed by LC-MS. The reactions were terminated by the addition of one volume of methanol and the samples were vortexed and centrifuged prior to separation on an Agilent 1290 Infinity II HPLC coupled to an Agilent 6495 Triple Quad mass spectrometer equipped with an electrospray ionization source (LC-QQQ; Agilent Technologies, CA, USA). An Agilent InfinityLab Poroshell 120 SB-C18 column (2.1 × 100 mm, 2.7 µm) was used at a controlled temperature of 30 °C and 5 µL of each reaction mixture was injected on the column. Anthocyanin and flavonols were separated using a gradient of 5 % formic acid in water (eluent A) and acetonitrile (eluent B) at a flow rate of 0.3 mL/min, as follows: 5 % to 90 % B (0–19 min), 90 % to 100 % B (19–21 min), and 5 % B (21–24 min). Mass spectra were recorded using multiple reaction monitoring (MRM) and positive ionisation mode. The MS source parameters and fragment ions are shown in Supplementary Table S3. All data were processed using the MassHunter qualitative analysis software version 10.0 (Agilent Technologies, CA, USA). Reactions involving phenolic acid substrates were analysed as described above, except for the use of 0.1 % formic acid as eluent A and negative ionisation mode to achieve better detection sensitivity of the targeted phenolic acid glycoconjugates.

### 2.8 Statistical analyses

Gene expression data obtained by qRT-PCR analysis, fruit weight and total soluble solids data were analysed using a one-way analysis of variance test (ANOVA) coupled to a Waller-Duncan post hoc test using IBM SPSS statistical software. Significant differences in the UGT plate-reader assays were also calculated using ANOVA followed by Duncan’s multiple-range test at the 5 % level (*p* < 0.05).

### 2.9 Modelling and molecular dynamics simulations

A high-quality homology model of *Pa*UGT1 was constructed in ICM-Pro using the crystal structure of UDP-Glc flavonoid 3-*O*-glycosyltransferase from *Vitis vinifera* (*Vv*GT1; PDB: 2C1Z) and refined with 100 iterations. UDP-Glc was subsequently docked into the model using ICM-Pro. For molecular dynamics (MD) simulations, the missing residues of *Vv*GT1 were modelled in ICM-Pro, and the catalytically inactive uridine-5′-diphosphate-2-deoxy-2-fluoro-α-d-glucose molecule was replaced with UDP-Glc. Triplicate 500 ns MD simulations were performed for *Pa*UGT1 and *Vv*GT1 in the presence and absence of UDP-Glc in explicit solvent using the Charmm-27 forcefield in Gromacs and the TIP3 water model (Mackerell Jr et al., 2004, Van Der Spoel et al., 2005). Ligand topologies were generated using the SwissParam server (Zoete et al., 2011). Structures were centred in a dodecahedral box at least 10 Å from the periodic edge boundary. The system was solvated with the TIP3P water model, and sodium and chloride ions added to neutralise net charges of the system, which was energy minimised using the steepest descent algorithm until F_max_ < 1000 kJ/mol. To appropriately equilibrate the system, 1 ns restrained NVT and NPT simulations were performed with Particle Mesh Ewald (PME) electrostatics and Berendsen thermostat coupling at 300 K. Following equilibration, restraints were removed and three independent production simulations were performed for 500 ns. To generate equilibrated structures of *Pa*UGT1 and *Vv*GT1, the atom coordinates of the proteins and UDP-Glc over the last 10 ns coordinates were averaged from a single trajectory. To determine predicted binding modes of the acceptors cyanidin, kaempferol and ferulic acid to equilibrated *Pa*UGT1, acceptors were docked to the binding pocket in ICM-Pro and then refined. All MD analyses were performed using the *MDTraj* library (McGibbon et al., 2015).

## 3 Results

### 3.1 Identification of putative cherry *UGT* genes involved in anthocyanidin glycosylation

**Fig. 1:**
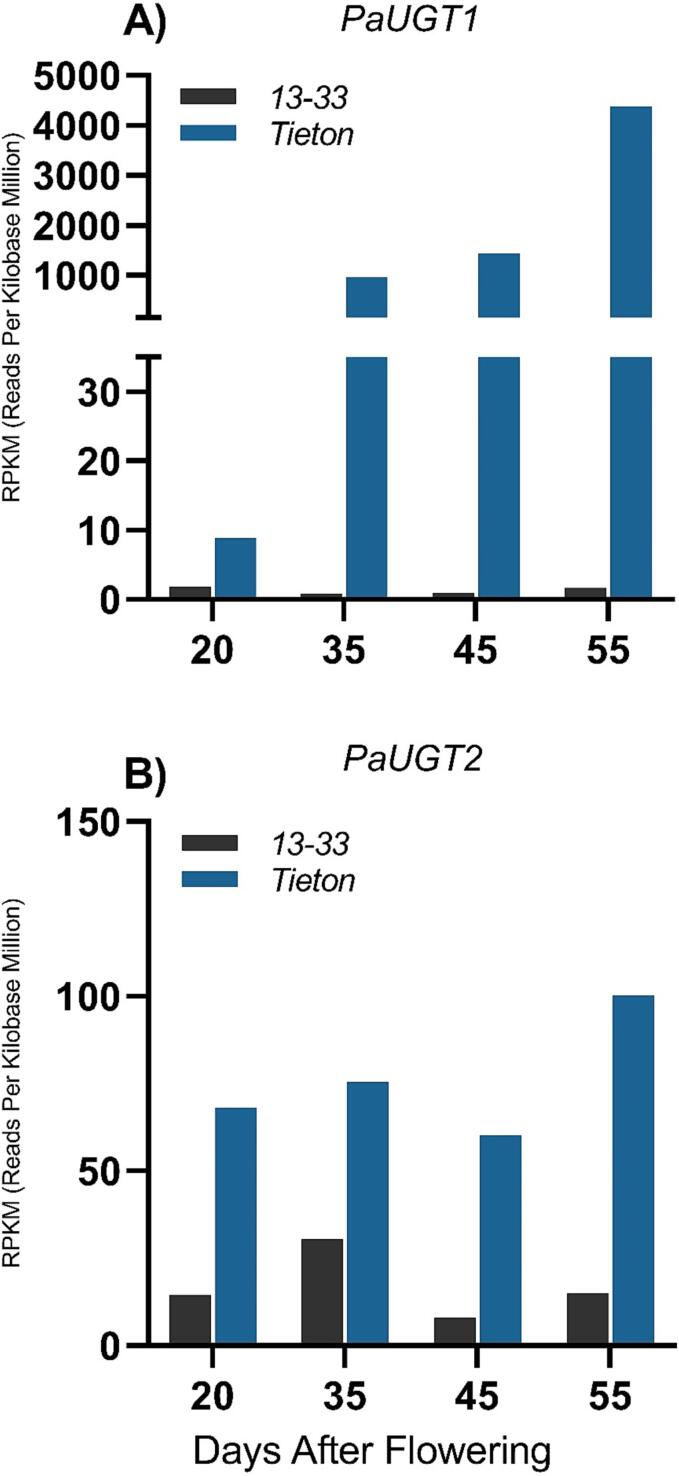
Differential expression of two putative cherry glycosyltransferases (*Pa*UGT1 and *Pa*UGT2) in two different cherry cultivars. The Tieton cultivar (red skinned) contains anthocyanins with corresponding biosynthetic genes highly expressed whereas the 13–33 cultivar (yellow skinned) is anthocyanin-deficient with little or no expression of these genes. Graphs represent transcript differences between the four stages of fruit ripening: 20 DAF (days after flowering), 35 DAF, 45 DAF and 55 DAF using digital gene expression (DGE) profiling (Wei et al., 2015). (For interpretation of the references to colour in this figure legend, the reader is referred to the web version of this article.)

As no UGT has been characterized in cherry, we sought to identify and analyze members of this class of enzymes involved in the glycosylation of anthocyanidins in this fruit. To this end, we first screened published RNA-Seq data (Wei et al., 2015) using the Pfam PF00201 domain, a signature of enzymes responsible for the glycosylation of diverse secondary metabolites, including phenolic compounds. Of the 154 sequences identified, the *PaUGT1* gene showed high expression levels in the anthocyanin-rich cultivar Tieton compared to the anthocyanin-deficient cultivar 13–33 (Fig. 1), which is consistent with data from earlier reports (Jin et al., 2016, Liang et al., 2020, Liu et al., 2013, Shen et al., 2014, Starkevič et al., 2015, Zhang and Zhu, 2023). RNA-seq data revealed that the transcript read numbers of *PaUGT1* increased significantly across development in the Tieton cultivar, with a 107-fold increase from 20 DAF to 35 DAF, 1.5-fold increase from 35 DAF to 45 DAF and a 3-fold increase from 45 DAF to 55 DAF (Fig. 1). Compared to the 13–33 cultivar, *PaUGT1* expression was 2574-fold higher in the Tieton cultivar at 55 DAF, indicating that this gene is turned off or significantly downregulated in the yellow-skinned cherry fruit and highly expressed in the red-skinned Tieton cultivar. For this reason, the *Pa*UGT1 was selected for further characterization as a putative anthocyanin glycosylating enzyme. *PaUGT2* was also selected as a gene of interest due to its significantly higher level of expression in the Tieton cultivar compared to the 13–33 fruit. However, transcript abundance for this gene showed little variation throughout fruit development compared to *Pa*UGT1 (Fig. 1). In addition, the expression of *PaUGT2* was much lower than that of *PaUGT1* as reflected by the Y axis scales in Fig. 1, suggesting that *Pa*UGT1 is the primary UGT responsible for the glycosylation of anthocyanins, and possibly other phenolic compounds, in sweet cherry.

Sequence alignments of *Pa*UGT1, *Pa*UGT2 and the grape *Vv*GT1 enzyme used for protein modelling revealed the presence of the so-called ‘Plant Secondary Product Glycosyltransferase’ (PSPG) motif in all three sequences. (Supplementary Fig. S1 and S2). Further sequence analysis and comparisons with proteins in public databases using the BLASTX algorithm, The Arabidopsis Information Resource (TAIR) and Krypto Encyclopedia of Genes and Genomes (KEGG) databases, provided further evidence that *Pa*UGT1 and *Pa*UGT2 are most likely UDP-glycosyltransferases involved in the biosynthesis of glycoconjugates of phenolic compounds. Indeed, the TAIR annotation suggested that the *PaUGT1* gene encodes an anthocyanidin 3-*O*-glucosyltransferase which specifically glucosylates the 3′-OH group of the flavonoid C-ring of substrates such as cyanidin, pelargonidin, kaempferol and quercetin. Results from these comparative *in silico* analyses also suggested that *Pa*UGT1 and *Pa*UGT2 are inverting enzymes with a GT-B fold, typical of members of the GT1 family in the CAZy Database (https://www.cazy.org). In conclusion, these observations strongly suggest that the candidate *PaUGT1* and *PaUGT2* genes are encoding GT1 UGTs involved in the biosynthesis of phenolic compounds in sweet cherry.

### 3.2 qPCR analysis of *PaUGT1*, *PaUGT2* and other genes involved in anthocyanin biosynthesis across sweet cherry fruit development

**Fig. 2:**
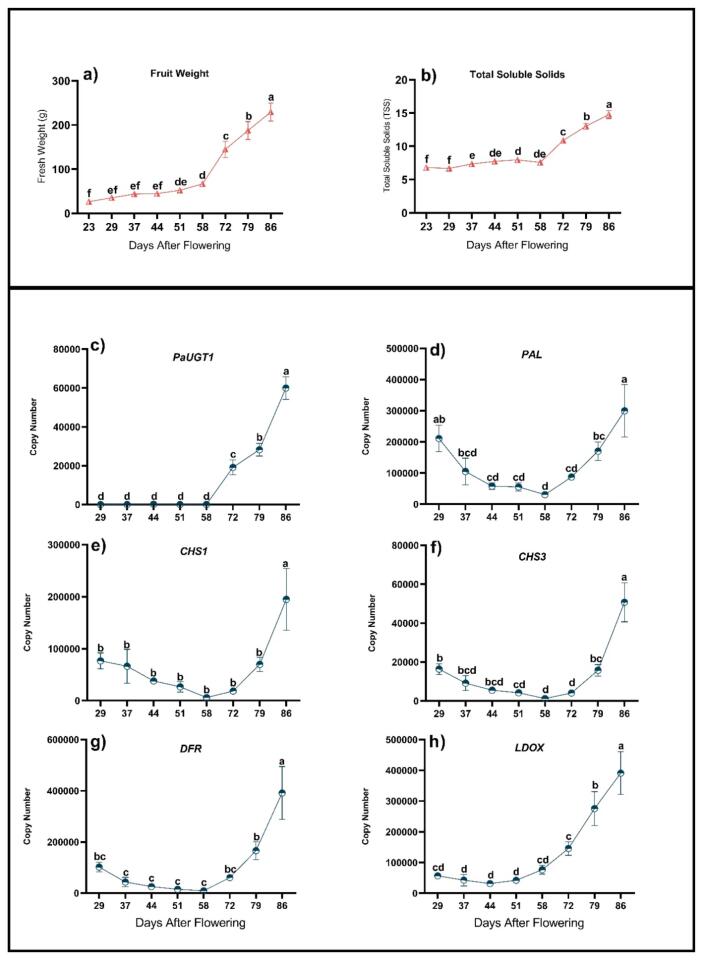
Fruit parameter measurements and qRT-PCR analysis of anthocyanin biosynthetic and regulatory genes in sweet cherry (Lapins cultivar) at eight timepoints during fruit development. a) fruit weight, b) total soluble solids, c) *PaUGT1,* d) *PAL*, e) *CHS1*, f) *CHS1*, g) *LDOX* and h) *DFR.* The data represent means ± standard error (*n* = 3); graphs are denoted by a different letter (a, b, c, d, e, f) if the means differ significantly (*p* < 0.0001) using one-way ANOVA followed by Waller-Duncan’s post hoc test.

In the next step of our work, we aimed to establish a link between anthocyanin production during fruit ripening and the expression of relevant genes involved in anthocyanin biosynthesis, including the putative UGT-encoding genes *PaUGT1* and *PaUGT2*. For this purpose, qRT-PCR analysis of gene expression was combined with weight and total soluble solids (TSS) measurements in sweet cherry fruits from the Lapins cultivar collected at different developmental stages. Significant increases in fruit weight and TSS marked 58 DAF as the entry point into the ripening phase (Fig. 2a, b) (Blazkova et al., 2002). qPCR analysis performed on *PaUGT1*, *PaUGT2* and five anthocyanin biosynthetic genes (*PAL, CHS1, CHS3, DFR* and *LDOX*) revealed that all genes, except *PAL*, *PaUGT1* and *PaUGT2*, were expressed at low levels early in fruit development (Fig. 2c-h). The expression of *PaUGT2* was too low throughout developmental stages of the Lapins cultivar to be reliably quantified, suggesting varietal differences in expression levels of this gene. *PaUGT1* followed a similar expression pattern as *CHS1*, *CHS3*, *DFR* and *LDOX*, with no or relatively low expression during 29–58 DAF. Compared to the other genes, the expression of the *PAL* gene decreased from a significantly high level at 29 DAF to a minimum at 58 DAF. From this stage, the expression of all genes, apart from *PaUGT2*, followed a similar trend, with a steady increase throughout the latest developmental stages analyzed (Fig. 2c-h). These data are consistent with the pattern of anthocyanin accumulation typically observed during ripening of sweet red cherry cultivars (Clayton-Cuch et al., 2021, Jin et al., 2016, Liu et al., 2013, Shen et al., 2014, Starkevič et al., 2015, Zhang and Zhu, 2023) and confirm the published RNA-Seq data from Wei et al. (2015). They also support the involvement of *PaUGT1* in anthocyanin biosynthesis during the ripening phase (Fig. 1, Fig. 2).

### 3.3 Identification of the phenolic compounds synthesized in Lapins cherry fruit

Expression data suggested that *Pa*UGT1 and *Pa*UGT2 are involved in the biosynthesis of anthocyanins (Fig. 1, Fig. 2). However, many UGTs from the GT1 family have been established to glycosylate more than one phenolic compound (https://www.cazy.org). Thus, fruits entering ripening phase and showing readily detectable expression of *PaUGT1* (i.e., fruits at 72 DAF; Fig. 2**c**) were analyzed by HPLC-QTOF to identify the glycosylated phenolic compounds present and select relevant substrates for further enzyme characterization. As shown in Supplementary Table S4, 38 phenolic compounds were tentatively identified through molecular formula prediction based on accurate mass, MS/MS fragmentation patterns and retention behaviour on reverse-phase C18 column chromatography, as well as comparisons with published literature (Martini et al., 2017). Based on their structural properties, the identified phenolic compounds were categorized into four groups: hydroxycinnamic acids, flavonols, hydroxybenzoic acids and anthocyanins. A total of 18 hydroxycinnamic acids were identified, of which one third were glycosylated: coumaric acid hexoside, caffeic acid hexoside, ferulic acid hexoside, and three caffeoylquinic acid hexoside isomers. Six of the 12 identified flavonols were glycosylated derivatives of quercetin and kaempferol, which carried rutinoside, hexoside, or both types of conjugation. Moreover, all hydroxybenzoic acids in cherry extract contained hexoside groups, and all anthocyanins were glycosylated with either hexoside or rutinoside moities. The anthocyanins found in Lapins cherry fruit were cyanidin-rutinoside, cyanidin-glucoside, peonidin-rutinoside and pelargonidin-rutinoside (Supplementary Table S4). These were also reported previously in other cherry species (Martini et al., 2017). Based on these results, anthocyanins, flavonols and phenolic acids were used as substrates for the enzymatic assays of recombinant *Pa*UGT1 and *Pa*UGT2 to determine their specificity.

### 3.4 Biochemical characterization of recombinant *Pa*UGT1 and *Pa*UGT2

**Fig. 3:**
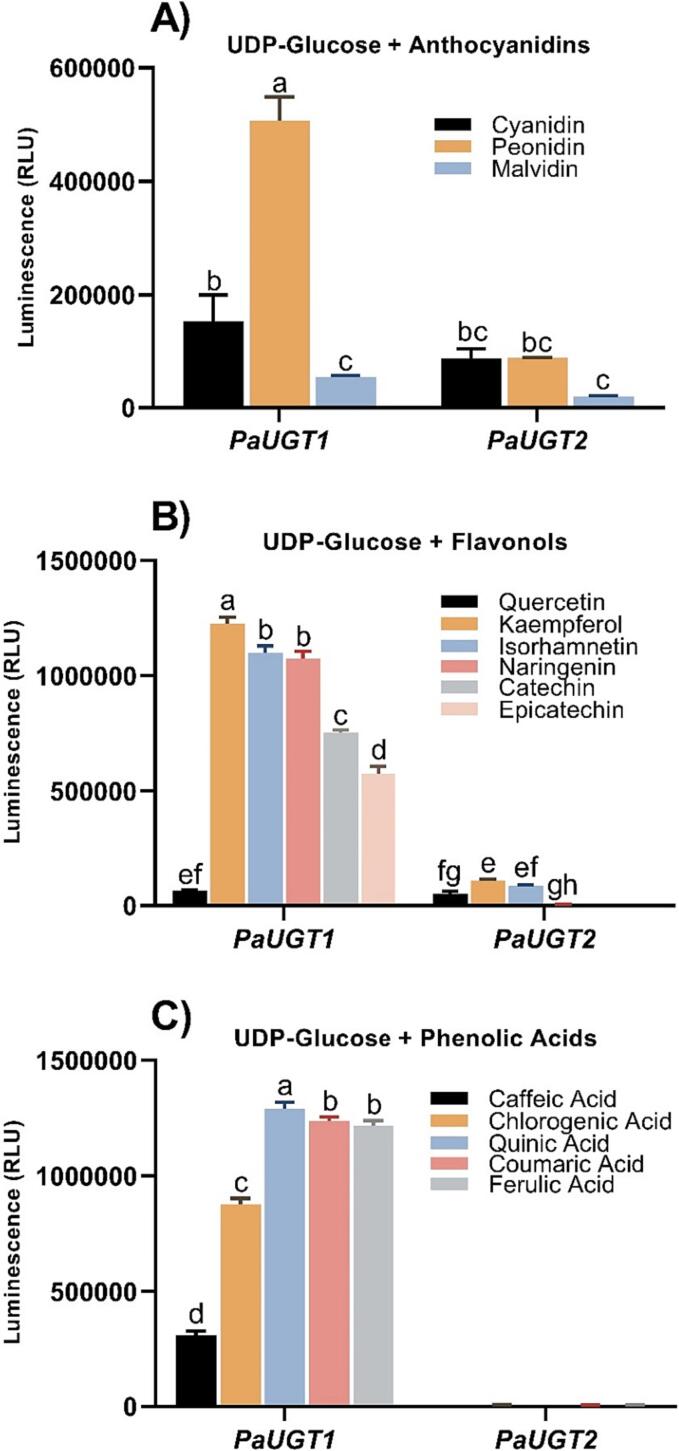
Recombinant *Pa*UGT1 and *Pa*UGT2 enzymes assayed in the presence of a broad range of sugar acceptors. The level of activity was determined by quantifying the amount of UDP released during the reaction, which was measured using the coupled enzyme UDP-Glo Glycosyltransferase Assay Kit. A) UDP-Glc and anthocyanidins, B) UDP-Glc and flavonols, C) UDP-Glc and phenolic acids. The data represent means ± standard error (*n* = 3); graphs are denoted by a different letter (a, b, c, d, e, f) if the means differ significantly (*p* < 0.0001) using one-way ANOVA followed by Waller-Duncan’s post hoc test.

*Pa*UGT1 and *Pa*UGT2 were recombinantly expressed as His-tagged versions in *E. coli* and purified (Supplementary Fig. S3), and their catalytic activity was assessed on a diversity of substrates using the UDP-Glo™ Glycosyltransferase Assay Kit. Both proteins were assayed using UDP-Glc and UDP-Gal as sugar donors, and 15 sugar acceptor molecules from different classes of phenolic compounds. The purified recombinant *Pa*UGT1 protein was active on a broad range of acceptors, with significant glucosyl transfer activity on anthocyanidins (cyanidin, peonidin and malvidin), flavonols (quercetin, kaempferol, isorhamnetin, naringenin, catechin and epicatechin) and phenolic acids (caffeic, chlorogenic, quinic, coumaric and ferulic acids) (Fig. 3). When UDP-Glc was replaced by UDP-Gal the level of activity measured was highly reduced and hardly detectable in many instances (compare Y axis scales in Fig. 3 and Supplementary Fig. S4), indicating that *Pa*UGT1 is primarily a UDP-glucosyltransferase with minimal UDP-galactosyltransferase activity. Similar to *P*aUGT1, the recombinant *Pa*UGT2 protein displayed glucosyltransferase activity toward a diverse range of phenolic aglycones from the flavonol and anthocyanidin families, but it was inactive on phenolic acids (Fig. 3). This enzyme was also able to use UDP-Gal as a sugar donor, but the activity detected was much lower than in the presence of UDP-Glc (Fig. 3 and Supplementary Fig. S4). In addition, both glucosyl and galactosyl transferase activities of *Pa*UGT2 were significantly lower compared to *Pa*UGT1.

In summary these data show that *Pa*UGT1 and *Pa*UGT2 are predominantly UDP-glucosyltransferases, with *Pa*UGT1 displaying significantly higher activity compared to *Pa*UGT2. The capacity of these UGTs to utilise two (*Pa*UGT2) or three (*Pa*UGT1) different classes of phenolic compounds demonstrates their promiscuity in terms of sugar acceptors, which suggests their involvement in the formation of multiple glycoconjugates *in planta*. As *Pa*UGT1 displayed higher and readily detectable activity well above background levels, in addition to broader substrate specificity, we focused further analyses on this enzyme.

**Fig. 4:**
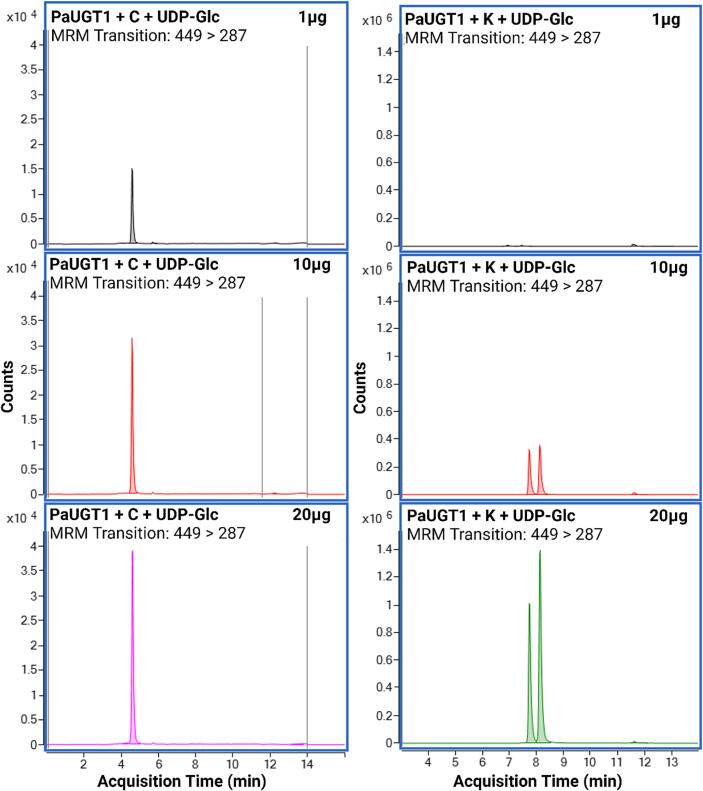
LCMS-QQQ data confirming catalytic activity of *Pa*UGT1 on an anthocyanidin (cyanidin or C) and a flavonol (kaempferol or K). Reaction conditions were as follows: 100 µL total volume containing 500 µM sugar acceptor, 1 mM sugar donor in 50 mM Tris–glycine buffer pH 8.0. All reactions were left for 1 h to incubate at room temperature with either 1, 10 or 20 µg of *Pa*UGT1 protein added, and an equal volume of 100 % MeOH was added to terminate the reaction. All reaction products were analyzed on an LCMS-QQQ instrument in positive ionisation and multiple reaction monitoring (MRM) mode.

**Fig. 5:**
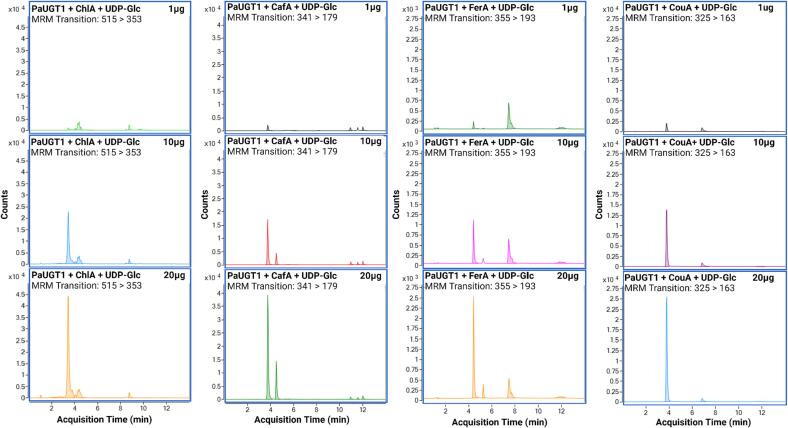
LCMS-QQQ data confirming catalytic activity of *Pa*UGT1 on four phenolic acid substrates, i.e., chlorogenic acid (ChlA), caffeic acid (CafA), ferulic acid (FerA) and coumaric acid (CouA). Reaction conditions were as follows: 100 µL total volume containing 500 µM sugar acceptor, 1 mM sugar donor in 50 mM Tris–glycine buffer pH 8.0. All reactions were left for 1 h to incubate at room temperature with either 1, 10 or 20 µg of *Pa*UGT1 protein added, and an equal volume of 100 % MeOH was added to terminate the reaction. All reaction products were analyzed on an LCMS-QQQ instrument in negative ionisation and multiple reaction monitoring (MRM) mode.

To demonstrate the identity of the products formed by *Pa*UGT1, assays were repeated as presented above, and the reaction mixtures recovered after one h incubation in the presence of increasing amounts of protein were analyzed by LCMS-QQQ in MRM mode (Fig. 4, Fig. 5; Supplementary Fig. S5). Representative catalytic reactions of *Pa*UGT1 incubated with UDP-Glc and either cyanidin or kaempferol are presented in Fig. 4. The addition of increasing amounts of *Pa*UGT1 in the reaction mixtures resulted in larger glycoconjugate peaks, demonstrating that the protein mediates glucosyl transfer in a concentration-dependent manner and that the reactions are indeed catalytic (Fig. 4). Interestingly, the chromatograms obtained when kaempferol was used as a substrate revealed the formation of two kaempferol glycoconjugates that exhibit slightly different retention times, suggesting that two isomers of the same product are formed by *Pa*UGT1.

In addition to the anthocyanin cyanidin and the flavonol kaempferol, *Pa*UGT1 also catalysed the glycosylation of phenolic acids in a concentration-dependent manner, as shown in Fig. 5 with chlorogenic, caffeic, ferulic and coumaric acids. Glycoconjugates of these compounds naturally occur in the sweet cherry Lapins fruit (Supplementary Table S4) as well as in and other cherry cultivars (Faienza et al., 2020, Hu et al., 2021, Martini et al., 2017). Similar to the kaempferol reaction (Fig. 4), dual peaks were observed at the end of the reactions containing caffeic and ferulic acids as substrates (Fig. 5), which also suggest the formation of two different glycoconjugate isomers.

### 3.5 *In silico* structure prediction of *Pa*UGT1 and modelling of enzyme/substrate complexes

**Fig. 6:**
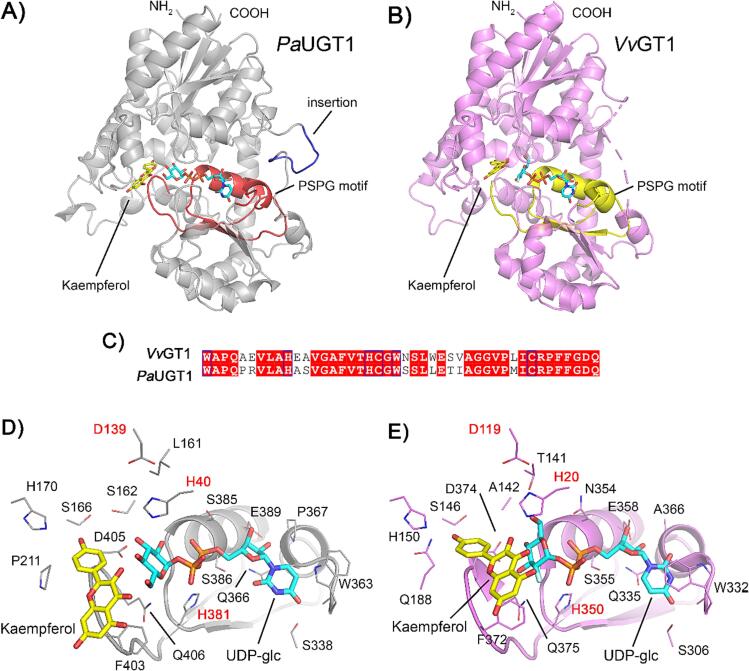
*Pa*UGT1 likely utilises the conventional GT-B catalytic transfer mechanism. A) Equilibrated model of *Pa*UGT1 complexed with UDP-Glc (cyan) and Kaempferol (yellow). The PSPG motif is coloured in red and the eight-amino-acid insertion is coloured in blue. B) Crystal structure of *Vv*GT1 complexed with a catalytically inactive structural analogue of UDP-Glc, and kaempferol (PDB: 2C1Z); the PSPG motif is coloured in yellow. C) Sequence alignment of the PSPG motif from *Vv*GT1 and *Pa*UGT1 demonstrates high conservation between residues. D-E) Interactions with the PSPG motif, UDP-Glc (cyan) and Kaempferol (yellow) for *Pa*UGT1 (D) and *Vv*GT1 (E); residues involved in the catalytic transfer of the glucosyl residue to the acceptor are labelled in bold red font. (For interpretation of the references to colour in this figure legend, the reader is referred to the web version of this article.)

In the absence of an experimentally determined crystal structure of *Pa*UGT1, a robust equilibrated structural model of the protein was generated in complex with UDP-Glc, using homology modelling and molecular dynamics simulations (Fig. 6). The crystal structure of the *Vv*GT1 enzyme from *Vitis vinifera* L. (PDB ID: 2C1Z) in complex with kaempferol and uridine-5′-diphospho-2′-fluoro-glucose, a UDP-Glc substrate analogue, was utilised as a structural template (Offen et al., 2006). Consistent with the *Vv*GT1 structure, the *Pa*UGT1 model shows that the protein adopts a typical GT-B glycosyltransferase fold (Fig. 6A and 6B) and contains the N- and C-terminal Rossman folds indicative of GT1 family enzymes that glycosylate diverse polyphenolic compounds (Lairson et al., 2008). Over the duration of each simulation performed in the presence and absence of UDP-Glc, the root-mean squared fluctuation (RMSF) of each residue converged with that of *Vv*GT1, suggesting comparable tertiary dynamics (Supplementary Fig. S6). Compared to *Vv*GT1, the cherry protein is characterized by an N-terminal extension of 20 amino acids and an insertion of a segment of 8 amino acids between residues 252 and 253. The effect of this insertion on function is unclear, but given its proximity to the UDP-binding site it may play a role in the coordination of UDP-Glc in the active site of *Pa*UGT1. Each protein contains a PSPG motif of 44 amino acids, which is part of the core of the C-terminal Rossman fold and differs between the two proteins by 9 residues only (Fig. 6C).

Interactions between amino-acid sidechains and substrates are important for enzymatic activities. In our equilibrated model, the sidechain of W363 (W332 in *Vv*GT1) does not stack above the pyrimidine group of the sugar donor, but it is flipped by ∼180°, potentially sequestered by the nearby 8-amino-acid insertion (Fig. 6D and 6E). Consequentially, the UDP moiety is also flipped, resulting in weak hydrogen bonding with S338 and Q366 (Fig. 6D). Although interactions with the nucleoside diphosphate group of UDP-Glc are conserved, the orientation of the glucosyl residue significantly differs between *Pa*UGT1 and *Vv*GT1. Indeed, in *Vv*GT1 the 6-hydroxymethyl group of the glucosyl residue faces upward and hydrogen bonds with T141, whereas in *Pa*UGT1 the sugar moity and its C6 primary alcohol are rotated almost 180° and face downward (Fig. 6D and 6E). This different orientation is imposed by the proximity of a leucine at position 161 and a serine at position 162 in *Pa*UGT1 instead of a threonine and an alanine at positions 141 and 142 in *Vv*GT1 (Fig. 6D and 6E). In both proteins, however, the catalytic residues H20 and D119 of *Vv*GT1, and H40 and D139 of *Pa*UGT1, are optimally positioned for deprotonation of the acceptor and subsequent nucleophilic attack of the sugar donor, thereby catalysing transfer of the glucosyl moiety from UPD-Glc to the acceptor (Hiromoto et al., 2015). Importantly, the distance between the anomeric C atom of the glucosyl and H20/40 residues deviates by less than one Å between *Pa*UGT1 and *Vv*GT1. In both proteins, the sugar acceptor hydrogen bonds with a conserved histidine at position 150 in *Vv*GT1 and 170 in *Pa*UGT1, thereby anchoring the acceptor’s hydroxyl group (Fig. 6D and 6E). When bound, the sugar acceptor is positioned according to the orientation of the glucosyl residue, resulting in the distance between the 3′-hydroxyl group of the kaempferol acceptor and C1 of the glucosyl residue deviating by 2.1 and 2.9 Å, respectively, between *Pa*UGT1 and *Vv*GT1. All other residues which interact with the acceptor are conserved, aside for a proline residue at position 211 in *Pa*UGT1, which in *Vv*GT1 is a glutamine (Q188) (Fig. 6D and 6E). Substitution for a proline removes a hydrogen bond with the phenolic group of the sugar acceptor and increases the volume of the local environment, contributing to acceptor substrate promiscuity. Despite the relatively minor structural differences presented above, the *in silico* and experimental data indicate that the catalytic mechanism of *Pa*UGT1 is similar to that of other GT1 enzymes (Hiromoto et al., 2015).

## 4 Discussion

Our biochemical data unequivocally demonstrate that the *Pa*UGT1 and *Pa*UGT2 proteins from sweet cherry are UGTs capable of glycosylating anthocyanidins and flavonols, with *Pa*UGT1 also active on phenolic acids. To our knowledge, these enzymes are the first cherry UGTs that have been experimentally characterized following heterologous expression and purification. Their ability to glycosylate three different classes of phenolic compounds is also not common. Indeed, a relatively small number of glycosyltransferases have been shown to be active on diverse polyphenolic substrates. For example, four strawberry (*Fragaria x ananassa*) enzymes designated UGT71A33, UGT71A34, UGT71A35 and UGT71W2, are able to glycosylate flavonols, flavan-3-ols, anthocyanidins and phenolic acids, as well as the plant hormone abscisic acid (Song et al., 2015). Another study showed that a UGT from the flowering plant *Lobelia erinus* is active on diverse anthocyanidins, flavones and flavonols such as delphinidin, cyanidin, pelargonidin, malvidin, apigenin and quercetin (Hsu et al., 2017). More recently, we reported the characterization of a galactosyltransferase from red-skinned apple fruit (*Malus domestica* L.) with dual activity on flavonol and anthocyanidin substrates (Clayton-Cuch et al., 2023). Contrasting with this promiscuity with respect to the sugar acceptor substrates, UGTs typically display high specificity toward the sugar donors. UDP-Glc and UDP-Gal are the most common substrates involved in phytonutrient glycosylation, although other nucleotide-sugars are also utilised by different UGTs, such as UDP-xylose, UDP-rhamnose, UDP-arabinose and UDP-glucuronic acid (Alseekh et al., 2020). In our work, UDP-Glc and UDP-Gal were tested as these are the sugar donors corresponding to the flavonoid glycoconjugates that occur in sweet cherry (He et al., 2022, Zhang et al., 2020).

To elucidate the molecular determinants of substrate specificity, we undertook the modelling of the *Pa*UGT1 structure (Fig. 6). The protein adopts a typical GT-B fold, encompassing both the N- and C-terminal Rossman folds which are conserved in GT1 family enzymes responsible for the glycosylation of phenolic compounds (He et al., 2022). In addition, the PSPG motif, an amino-acid sequence spanning 44 residues within the C-terminal domain of UGTs, is also present in *Pa*UGT1 and *Vv*GT1 (Fan et al., 2017). This motif exhibits a high degree of conservation among enzymes belonging to the GT1 family and serves as a pivotal site for catalytic residues (Fan et al., 2017). Notably, the residues responsible for the catalytic transfer of the glucosyl moiety to the O3 hydroxyl group in ring C of the acceptor molecule exhibit a similar arrangement in *Pa*UGT1, *Vv*GT1 and other reported UGTs (Hiromoto et al., 2015, Offen et al., 2006). However, in contrast to UGTs with established experimental structures, the sugar residue in *Pa*UGT1 adopts an unconventional orientation due to the absence of an essential stabilising interaction which occurs through T141 in *Vv*GT1, and a flip of the UDP pyrimidine group (Fig. 6). These changes likely confer flexibility to the glucosyl residue, thereby promoting the observed substrate promiscuity of *Pa*UGT1 with respect to the sugar acceptor. In addition, substrate selectivity might also be influenced by the presence of a proline at position 211 in *Pa*UGT1, which is a glutamine (Q188) in *Vv*GT1 (Fig. 6D, E). These structural differences highlight some of the potential mechanisms through which the typically promiscuous UGT enzymes can glycosylate a range of different substrates.

Anthocyanins are a particularly abundant class of phytonutrients in sweet cherry that require glycosylation to maintain their stability and solubility. Anthocyanin accumulation is typically low during early stages of fruit development and increases significantly thereafter, as has been reported in several studies (Karaaslan et al., 2016, Liu et al., 2013). Thus, the expression of the genes involved in anthocyanin biosynthesis is expected to follow a similar trend. Indeed, our qRT-PCR data showed a correlation between the levels of expression of the *PAL, CHS1, CHS3, DFR, LDOX,* and *PaUGT1* genes and the previously reported pattern of anthocyanin accumulation during fruit development (Clayton-Cuch et al., 2021, Karaaslan et al., 2016, Liu et al., 2013). In addition, the expression of these genes differed significantly between the red and yellow cherry fruits, further supporting their involvement in anthocyanin biosynthesis (Wei et al., 2015). Accordingly, the expression pattern of *PaUGT1* within different cherry cultivars has been reported in multiple studies, with transcript levels increasing significantly as the fruit ripens and anthocyanin concentrations increase (Clayton-Cuch et al., 2021, Jin et al., 2016, Liu et al., 2013, Shen et al., 2014, Starkevič et al., 2015, Zhang and Zhu, 2023). Interestingly, in some of these studies the expression of *PaUGT1* increased across development in both yellow-skinned and red-skinned cultivars of cherry (Jin et al., 2016, Liu et al., 2013). For example, compared to the Big Dragon and Rainier yellow cultivars, *PaUGT1* was expressed at a high level in the anthocyanin-rich Lapins cultivar, but the expression of the gene increased steadily across development of both yellow cultivars (Jin et al., 2016). This observation suggests that *PaUGT1* is involved in the biosynthesis of the other types of phenolic compounds produced in ripening cherry fruits, which is in agreement with our biochemical data showing that *PaUGT1* is active on a diversity of substrates, including flavonols, anthocyanins, flavan-3-ols and phenolic acids (Fig. 3, Fig. 4).

Understanding the substrate selectivity of UGTs could provide a new avenue for the modification of the phytonutrient composition of cherry and other fruits. Interestingly, however, previous studies aimed to alter the nutritional value of fruit by primarily targeting upstream transcription factors rather than downstream UGTs. For example, the first anthocyanin-enriched tomato fruit was produced in 2008 where two regulatory genes encoding a bHLH and MYB factor were overexpressed (Butelli et al., 2008). When these tomatoes enriched in anthocyanins were fed to cancer-susceptible Trp53 mice, the life span of the animals was significantly increased (Butelli et al., 2008). More recently, overexpression of a single *SIAN2* gene under the 35S promoter also produced a transgenic tomato that had increased anthocyanin concentration (Jian et al., 2019). Other genes involved in anthocyanin biosynthesis upstream of UGTs have also been used to engineer rice and maize plants with anthocyanin-rich endosperm (Liu et al., 2018, Zhu et al., 2017). In a recent study, a CRISPR system designed for transcriptional activation was used in pear to upregulate anthocyanin biosynthesis by targeting the promoters of *MYB10*, *MYB114*, *Bhlh3*, *DFR*, *ANS* and *UGT* (Ming et al., 2022). This led to the upregulation of several genes by at least 10-fold, leading to enhanced anthocyanin accumulation accompanied by a change of colour of the pear calli to red (Ming et al., 2022). This study indicates that when a *UGT* gene is included in the targets for upregulation, the concentration of glycosylated anthocyanins can increase to a greater extent (Ming et al., 2022). As glycosylation is the final step in the biosynthesis of many of these compounds, it may represent the rate limiting step. Considering that glycosylated forms of these phenolic compounds are present in the fruit at significantly higher concentrations than their aglycone counterparts, increasing the expression of rate-limiting UGTs should be pursued in future work. Here, we have identified and characterized a UGT enzyme in sweet red cherry fruit which is potentially the primary GT involved in the biosynthesis of many phenolic compounds, and therefore an ideal target for improving the phytonutrient composition of this fruit.

## 5 Conclusion

We report the characterization of *Pa*UGT1 and *Pa*UGT2, two UGT enzymes from sweet cherry, through heterologous expression in *E. coli*, purification, and biochemical demonstration of catalytic activity. Both enzymes are promiscuous in terms of sugar acceptor specificity, being able to glycosylate anthocyanidins and flavonols, as well as phenolic acids in the case of *Pa*UGT1. Both are primarily glucosyltransferases, but weaker galactosyltransferase activity was also detected. As the flavonoid glycoconjugates in sweet cherry are glucosyl and galactosyl derivatives, we propose that *Pa*UGT1, which shows higher activity *in vitro* than *Pa*UGT2 and whose expression levels correlate with anthocyanin biosynthesis during the ripening phase, is the primary enzyme involved in flavonoid glycosylation in this fruit. It has the potential to be employed to synthesize a diversity of glycoconjugates of flavonoids, thereby enhancing their stability and solubility before they are integrated into bioactive formulations. Additionally, its expression can be manipulated through breeding methods to boost the health-promoting properties of cherry fruit.

## Contributions

D.C-C conducted the experiments, analysed and interpreted the data, and wrote the first draft of the manuscript. L.Y. performed bioanalytical work and, together with D.B., provided further support and guidance for flavonoid analyses. D.M. and J.B.B performed computational modelling and structural analyses, and C.A.B. assisted with qPCR analyses. C.B. and V.B. coordinated experimental design, analysed and interpreted the data, wrote, and revised the manuscript. All authors have read and approved the final version of the manuscript.

## CRediT authorship contribution statement

**Daniel Clayton-Cuch:** . **Long Yu:** . **Daniel McDougal:** Investigation, Methodology, Writing – review & editing. **Crista A. Burbidge:** . **John B. Bruning:** Conceptualization, Methodology, Supervision, Writing – review & editing. **David Bradley:** . **Christine Böttcher:** Conceptualization, Formal analysis, Funding acquisition, Investigation, Methodology, Resources, Supervision, Validation, Writing – review & editing. **Vincent Bulone:** Conceptualization, Formal analysis, Funding acquisition, Project administration, Resources, Supervision, Validation, Writing – original draft, Writing – review & editing.

## Declaration of competing interest

The authors declare that they have no known competing financial interests or personal relationships that could have appeared to influence the work reported in this paper.

## Data Availability

Data will be made available on request.
