# Supplementary material for: Biochemical and in silico characterization of glycosyltransferases from red sweet cherry (Prunus avium L.) reveals their broad specificity toward phenolic substrates
Source: Food Chem (Oxf). 2023 Dec 31;8:100193. doi: 10.1016/j.fochms.2023.100193 (PMC10825616; doi:10.1016/j.fochms.2023.100193)
Supplement: Supplementary data 1 [file mmc1.docx]

**Supplementary Data (For Publication)**

**Biochemical and in silico characterization of glycosyltransferases from red sweet cherry (Prunus avium L.) reveals their broad specificity toward phenolic substrates**

Daniel Clayton-Cuch^a,b^, Long Yu^a,1^, Daniel McDougal^c^, Crista A. Burbidge^b^, John B. Bruning^c^, David Bradley^d^, Christine Böttcher^b,^* and Vincent Bulone^a,e, 1,^*

^a^ Adelaide Glycomics, University of Adelaide, School of Agriculture, Food and Wine, Waite Campus, Adelaide, South Australia, Australia;

^b^ CSIRO, Waite Campus, Glen Osmond, SA 5064, Australia

^c^ Institute for Photonics and Advanced Sensing, (IPAS), School of Biological Sciences, The University of Adelaide, Adelaide, South Australia 5005, Australia

^d^ Agilent Technologies Australia Pty Ltd, Mulgrave, Melbourne, Victoria, Australia

^e^ Division of Glycoscience, Department of Chemistry, School of Engineering Sciences in Chemistry, Biotechnology and Health, Royal Institute of Technology (KTH), AlbaNova University Centre, Stockholm, Sweden

^1^ Present address: College of Medicine and Public Health, Flinders University, Bedford Park Campus, Sturt Road, SA, 5042, Australia

***Corresponding authors:**Vincent Bulone
email: vincent.bulone@flinders.edu.au

Telephone: +61 (0)8 7221 8558

Christine Böttcher
email: christine.bottcher@csiro.au

**Table S1**. Gene-specific qPCR primers, with PCR product sizes (bp) and optimal acquisition temperatures for the genes analysed.

| **Gene** | **Forward Primer (5’-3’)** | **Reverse Primer (5’-3’)** | **Amplicon bp** | **Acquisition Temperature °C** |
| --- | --- | --- | --- | --- |
| *CHS1* | CAGGAGATGGACTGGATTGG | CGAACGAAACAGATGCATAAAA | 148 | 77 |
| *CHS3* | ATGTCGAGTGCTTGTGTGCTG | TACACCATGATGCATCTGGAAG | 285 | 78 |
| *LDOX* | CGAGTATGCTAAGGAACTGAGG | TGAGGGCAAACTGGGTAGTAG | 156 | 78 |
| *PaUGT1* | TGTTGAGGATGGGGTTTTTAC | AGTACAGCTCGGTTATTCTTATGC | 243 | 80 |
| *PaUGT2* | TCAGAGACAGAGCATTGAAGC | CGCAAGGCAGCAAAACTACA | 291 | 80 |
| *DFR* | CTTGGAGGACATGTTCGTAGG | AAAGAACCCAACAGACACTAATCC | 189 | 81 |
| *PAL* | ACAGCTATCTGCGAGGGAAA | CACAGTCTACCAGCAATGCAA | 165 | 79 |
| *EFA* | CTGGTGCTAAGATCACCAAGG | GCCTAACAATGACGACCAAAA | 185 | 78 |

**Table S2.** Coding sequences of the *PaUGT1* and *PaUGT2* genes.

| **Gene name** | **Sequence** |
| --- | --- |
| ***PaUGT1***  ***XM_021949698.1*** | ATGATCCCACTCCAATTAGTCTTATTAGCACATACATATATGGCACCACAACCGATTGATGATGATCATGTTGTGTATGAGCATCATGTGGCGGCCCTAGCCTTCCCTTTCTCCACCCATGCCAGTCCCACCCTTGCGCTCATCCGCCGCCTAGCCGCTGCCTCTCCCAACACTCTCTTCTCATTCTTCAGCACTTCACAATCCAACAACTCACTCTTTTCCAACACTAATACCAATCTTCCACGTAACATAAAGGTGTTTGATGTGGCTGATGGCGTCCCCGACGGCTATGTGTTTGCGGGTAAGCCCCAGGAGGATATTGAGCTTTTCATGAAGGCTGCCCCCCACAACTTTACAACCAGCTTAGACGCCTGCGTGGCTCACACCGGGAAGCGTCTCACCTGCTTGATCACCGACGCCTTCCTTTGGTTTGGGGCCCACTTGGCACACGACTTGGGAGTCCCTTGGCTCCCTCTTTGGCTCTCCGGACTTAATTCCCTCTCCCTCCATGTCCATACTGACCTCCTCCGCCACACCATCGGAACTCAAAGTATTGCAGGTCTTGAAAACGAACTCATCACCAAGAATGCCAACATCCCAGGAATGTCCAAAGTACGAATCAAAGATTTGCCTGAAGGTGTCATCTTTGGAAACTTGGACTCAGTCTTCTCACGCATGCTGCATCAAATGGGCCAACTGCTACCCCGTGCCAACGCAGTTCTCGTAAACAGCTTTGAAGAACTGGATATTACCGTAACAAACGATTTGAAATCCAAATTCAACAAGCTTCTCAATGTCGGACCTTTCAACCTAGCTGCTGCTGCTTCCCCTCCACTGCCGGAAGCCCTAACAGCCGTAGACGACGTTACTGGTTGCCTGTCCTGGCTTGACAAACAAAAGGCTGCATCCTCCGTGGTGTATGTTAGTTTTGGGTCAGTCGCAAGGCCACCGGAGAAGGAGCTTATGGCGATGGCACAAGCCTTGGAAGCCAGCGGTGTACCCTTCTTATGGTCTCTCAAGGACAGTTTCAAGACACCTTTGCTAAATGAGTTGCTAGTAAAAGCAAGTAATGGGATGGTGGTGCCCTGGGCTCCCCAGCCACGTGTCCTAGCCCATGCATCAGTCGGAGCCTTCGTAACGCACTGCGGTTGGAGCTCATTGCTGGAGACTATAGCAGGCGGGGTGCCAATGATTTGCAGGCCTTTCTTTGGCGACCAAAGGGTCAACGCAAGAACGGTGGAGGACGTGTTGGAGATCGGGGTCACTGTTGAGGATGGGGTTTTTACCAAGCACGGCTTGATCAAATATTTCGATCAAGTTTTGTCACAACAAAGAGGAAAGAAAATGAGAGGCAACATAAACACCGTTAAACTACTCGCACAACAGTCGGTTGAACCAAAAGGGAGCTCAGCTCAGAATTTCAAATTATTGCTAGATGTCATATCTGGATCCACTAAAGTATAA |
| ***PaUGT2***  ***XM_021958043.1*** | ATGGAGCAAAGCAGAGGCCTGAGATTGATACTTCTCCCACTGCCATTTCAAGGCCATATAAACCCCATGCTAGAACTGGGCAACCTTCTTCACTCCAAAGGCTTTTCCATAACCATCGTCCACACCAAGTTCAACTCTCTCAACCCTTCAAGCCACCCACACTTCACCTTCCACTCAATCCCTGTCGACTTATCTGAATCTGAGGCCTCCAAAAAGGATGCTGGCCGTCTTCTTTCTATTATAAACGCTAAATGTGTTGAGCCTTTCAGGGAATGCTTGGCTACCTTGTTATCCGATGCAGAGGAGGACCCTGTTGCTTGCTTGATCTCAGACCCTCTCCTTCACTTCACTCGATCAGTTGCAGAGAGCCTTGAGCTCCCAAGGATTCTGTTAAGGACTGGGGGCGCCGCTTCCTTTGCTGTTTATGCTGCTTTTCCACTTTTGAAGGAAAAGGGTTACCTCCCAATACAAGATTCTCGACTACAAGAGCCAGTGACAGAGCTGTCACCTTTCAAAGTTAAAGACCTGCCCAGAATGGACACTTGTGACCCTGAGATTTTTTACCAACTGATAACCAACATGGCAAATGAACCTAAGGCCTCATCTGGACTCATTTTCAACACTTTCGAAGACCTCGAACAACATGCACTTGCCACACTTAGCCAAGATTCTTACCCCAATATTCCAATTTTCCCAATAGGCCCATTTCACAAGTGTGACTTTGCAGCATCCTCTTCTTCAACTGGCTTATTAACAGAAGACCAGAGTTGCATTTCATGGCTAAACACTCAAGCACCGAAATCTGTTGTGTATGTTAGCTTTGGGAGCCTTGCTGCAGTAAAAGAAGCTCAGTTTTTGGAGATAGCTTGGGGACTAGCCAACAGCAACCACCCATTCTTGTGGGTGGTTCGACCCGGATTAGTCCATGGATCAGAACCATTGCCTGATGGGTTTCTTGAGAGTTTGAATGGGAGGGGCCACATTGTGAAATGGGCTCCACAGAAACAAGTACTGGCTCATCCAGCAGTTGGAGCCTTTTGGACTCACAATGGTTGGAATTCTACATTGGAGAGTGTTTGTGAGGGGGTCCCTATGATTTGCATGCCATGTTTCAGTGATCAAATGGCCAATGCAAGATATGTGAGCCATGTTTGGAAGATTGGGGTGCAGATAGAGAATGCCATTGAGAGAGCTGAGATTGAAAGAACCATTAATTTGTTAATGGTGGAGAAAGAAGGGGTAGAGATCAGAGACAGAGCATTGAAGCTAATGGAGAAGGCAAATCTTTGCCTCAAAGAAGGTGGCTCTTCATACCAATCTTTGGATAGATTGGTTAAACATATTTTATCAATAAAATCCTTTGCTTTTGAAAAGCAGAGTGAGTGA |

**Table S3*.*** Anthocyanin, flavonol and phenolic acid compounds with their transitions, fragmentor and collision energy voltages used in LC-MS/MS analysis.

| **Compound** | **GasTemp (°C)** | **GasFlow (L/min)** | **Nebulizer (psi)** | **Sheath Gas Heater (°C)** | **Sheath Gas Flow (L/min)** | **Capillary (V)** | **Charging (V)** | **Precursor ion**  **MS [M-H] +** | **Product**  **ion MS/MS** | **Fragmentor (V)** | **Collision energy (eV)** |
| --- | --- | --- | --- | --- | --- | --- | --- | --- | --- | --- | --- |
| Cyanidin-Glucoside | 230 | 20 | 40 | 300 | 12 | 3000 | 500 | 449.0 | 287.0 | 166 | 22 |
| Kaempferol-Glucoside | 230 | 20 | 40 | 300 | 12 | 3000 | 500 | 449.0 | 287.0 | 166 | 22 |
| Chlorogenic Acid-Glucoside | 230 | 20 | 40 | 300 | 12 | 3000 | 500 | 515.0 | 353.0 | 166 | 22 |
| Caffeic Acid-Glucoside | 230 | 20 | 40 | 300 | 12 | 3000 | 500 | 341.0 | 179.0 | 166 | 22 |
| Ferulic Acid-Glucoside | 230 | 20 | 40 | 300 | 12 | 3000 | 500 | 355.0 | 193.0 | 166 | 22 |
| Coumaric Acid-Glucoside | 230 | 20 | 40 | 300 | 12 | 3000 | 500 | 325.0 | 163.0 | 166 | 22 |

**Table S4.** Characterisation of phenolic compounds in Lapins cherry extract by LC-QTOF mass spectrometry

| **No** | **Compound Name** | **Molecular Formulation** | **Theoretical [M-H]^-^ (m/z)** | **Observed [M-H]^-^ (m/z)** | **Error (ppm)** | **MSMS product ion (m/z)** |
| --- | --- | --- | --- | --- | --- | --- |
|  | **Hydroxycinnamic acids** |  |  |  |  |  |
| 1 | 3-Caffeoylquinic acid *cis* | C_16_H_18_O_9_ | 353.0878 | 353.0879 | 0.28 | 191(100%), 179(65%), 135(30%) |
| 2 | 3-Caffeoylquinic acid *trans* | C_16_H_18_O_9_ | 353.0878 | 353.0879 | 0.28 | 191(100%), 179(63%), 135(28%) |
| 3 | 5-Caffeoylquinic acid | C_16_H_18_O_9_ | 353.0878 | 353.0879 | 0.28 | 191(100%) |
| 4 | 4-Caffeoylquinic acid | C_16_H_18_O_9_ | 353.0878 | 353.0879 | 0.28 | 173(100%), 179(70%), 191(53%), 135(40%) |
| 5 | 3-Coumaroylquinic acid | C_16_H_18_O_9_ | 337.0929 | 337.0954 | 7.42 | 163(100%), 119(38%), 191(18%) |
| 6 | 4-Coumaroylquinic acid | C_16_H_18_O_9_ | 337.0929 | 337.0941 | 3.56 | 173(100%), 163(22%) |
| 7 | 3-Feruloylquinic acid | C_17_H_20_O_9_ | 367.1034 | 367.1048 | 3.81 | 193(100%), 134(31%) |
| 8 | 5-Feruloylquinic acid *trans* | C_17_H_20_O_9_ | 367.1034 | 367.1053 | 5.18 | 161(100%) |
| 9 | 5-Feruloylquinic acid *cis* | C_17_H_20_O_9_ | 367.1034 | 367.1043 | 2.45 | 179(100%), 135(51%), 161(24%) |
| 10 | Caffeoylquinic acid-hexoside I | C_22_H_28_O_14_ | 515.1406 | 515.1426 | 3.88 | 179(100%), 341(14%), 353(7%) |
| 11 | Caffeoylquinic acid-hexoside II | C_22_H_28_O_14_ | 515.1406 | 515.1435 | 5.63 | 179(100%), 341(93%) |
| 12 | Caffeoylquinic acid-hexoside III | C_22_H_28_O_14_ | 515.1406 | 515.1435 | 5.63 | 179 (100%), 341(95%) |
| 13 | 3,5-Dicaffeoylquinic acid | C_25_H_24_O_12_ | 515.1195 | 515.1222 | 5.24 | 353(100%), 191(79%) |
| 14 | 4,5-Dicaffeoylquinic acid | C_25_H_24_O_12_ | 515.1195 | 515.1211 | 3.11 | 353(100%), 191(91%), 179(65%) |
| 15 | Caffeoylshikimic acid | C_16_H_16_O_8_ | 335.0772 | 335.0785 | 3.88 | 161(100%), 135 (29%) |
| 16 | Coumaric acid hexoside | C_15_H_18_O_8_ | 325.0929 | 325.0944 | 4.61 | 145(100%), 163(16%), 119(13%) |
| 17 | Caffeic acid hexoside | C_15_H_18_O_9_ | 341.0878 | 341.0892 | 4.10 | 179(100%), 135(28%) |
| 18 | Ferulic acid hexoside | C_16_H_2_0O_9_ | 355.1035 | 355.1047 | 3.38 | 193(100%), 149(42%), 134(42%), 178(40%) |
|  | **Flavonols** |  |  |  |  |  |
| 19 | Catechin | C_15_H_14_O_6_ | 289.0718 | 289.0735 | 5.88 | 245(100%), 205(67%) |
| 20 | Epicatechin | C_15_H_14_O_6_ | 289.0718 | 289.0737 | 6.57 | 245(100%), 205(67%) |
| 21 | Catechin hexoside | C_21_H_24_O_11_ | 451.1246 | 451.1268 | 4.88 | 137(100%), 271(18%) |
| 22 | Procyanidin dimer B I | C_30_H_26_O_12_ | 577.1352 | 577.1378 | 4.51 | 289(100%), 407(83%), 425(31%), 451(25%) |
| 23 | Procyanidin dimer B II | C_30_H_26_O_12_ | 577.1352 | 577.1378 | 4.51 | 289(100%), 407(97%), 425(40%), 451(16%) |
| 24 | Procyanidin dimer B III | C_30_H_26_O_12_ | 577.1352 | 577.1373 | 3.64 | 289(100%), 407(63%), 425(29%), 451(29%) |
| 25 | Quercetin-rutinoside | C_27_H_30_O_16_ | 609.1461 | 609.1493 | 5.25 | 301(100%) |
| 26 | Quercetin-hexoside | C_21_H_20_O_12_ | 463.0882 | 463.0898 | 3.46 | 301(100%) |
| 27 | Quercetin-hexoside-rutinoside | C_33_H_40_O_21_ | 771.1989 | 771.2005 | 2.07 | 609(100%) |
| 28 | Kaempferol-hexoside | C_21_H_20_O_11_ | 447.0933 | 447.0934 | 0.22 | 284(100%), 285(81%) |
| 29 | Kaempferol-rutinoside | C_27_H_30_O_15_ | 593.1512 | 593.1548 | 6.07 | 284(100%), 285(96%) |
| 30 | Kaempferol-hexoside-rutinoside | C_33_H_40_O_20_ | 755.2040 | 755.2053 | 1.72 | 593(100%), 447(32%), 285(19%) |
| 31 | Taxifolin-rutinoside | C_27_H_32_O_16_ | 611.1618 | 611.1653 | 5.73 | 285(100%), 475(54%), 485(8%), 501(12%), 241(100%), 303(31%) |
|  | **Hydroxybenzoic acids** |  |  |  |  |  |
| 32 | Protocatechuic acid- hexoside | C_13_H_16_O_9_ | 315.0722 | 315.0728 | 1.90 | 152(100%), 108(63%), 109(37%) |
| 33 | Hydroxybenzoyl hexose | C_13_H_16_O_8_ | 299.0772 | 299.0769 | -1.00 | 137(100%), 93(99%) |
| 34 | Vanillic acid-hexoside | C_14_H_18_O_9_ | 329.0878 | 329.0891 | 3.95 | 167(100%), 152 (33%), 123(41%), 108(35%) |
|  | **Anthocyanins^a^** |  |  |  |  |  |
| 35 | Cyanidin-glucoside | C_21_H_21_O_11_ | 449.1078 | 449.1076 | -0.45 | 287(100%) |
| 36 | Cyanidin-rutinoside | C_27_H_31_O_15_ | 595.1657 | 595.1705 | 8.06 | 287(100%), 449(5%) |
| 37 | Peonidin-rutinoside | C_28_H_33_O_15_ | 609.1814 | 609.1817 | 0.49 | 301(100%), 463(6%) |
| 38 | Pelargonidin-rutinoside | C_27_H_31_O_14_ | 579.1708 | 579.1710 | 0.35 | 271(100%), 433(6%) |

^a^ indicate [M]^+^ rather than [M-H]^-^

**
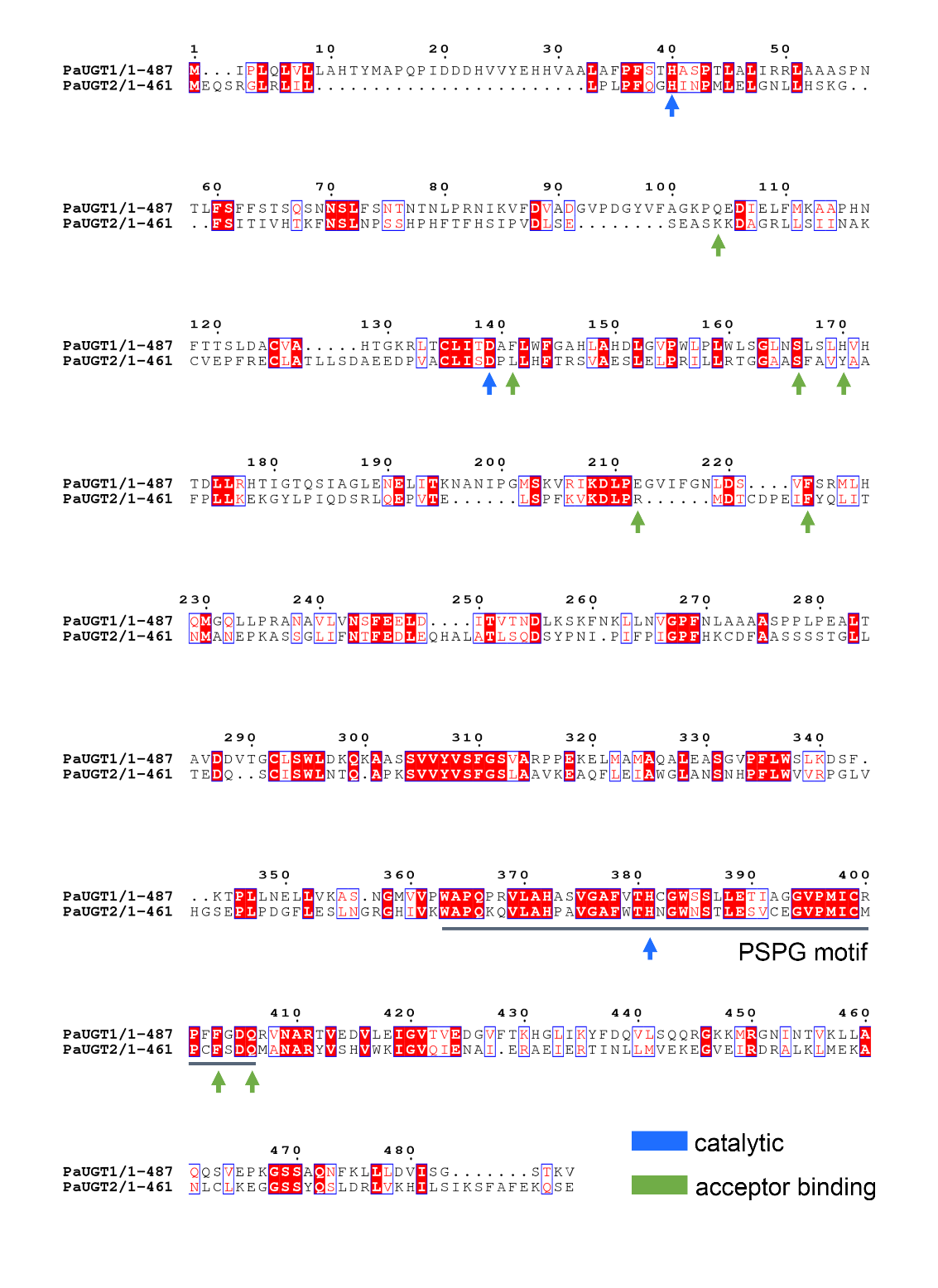
**

**Figure S1. Sequence alignment of *Pa*UGT1 and *Pa*UGT2.**


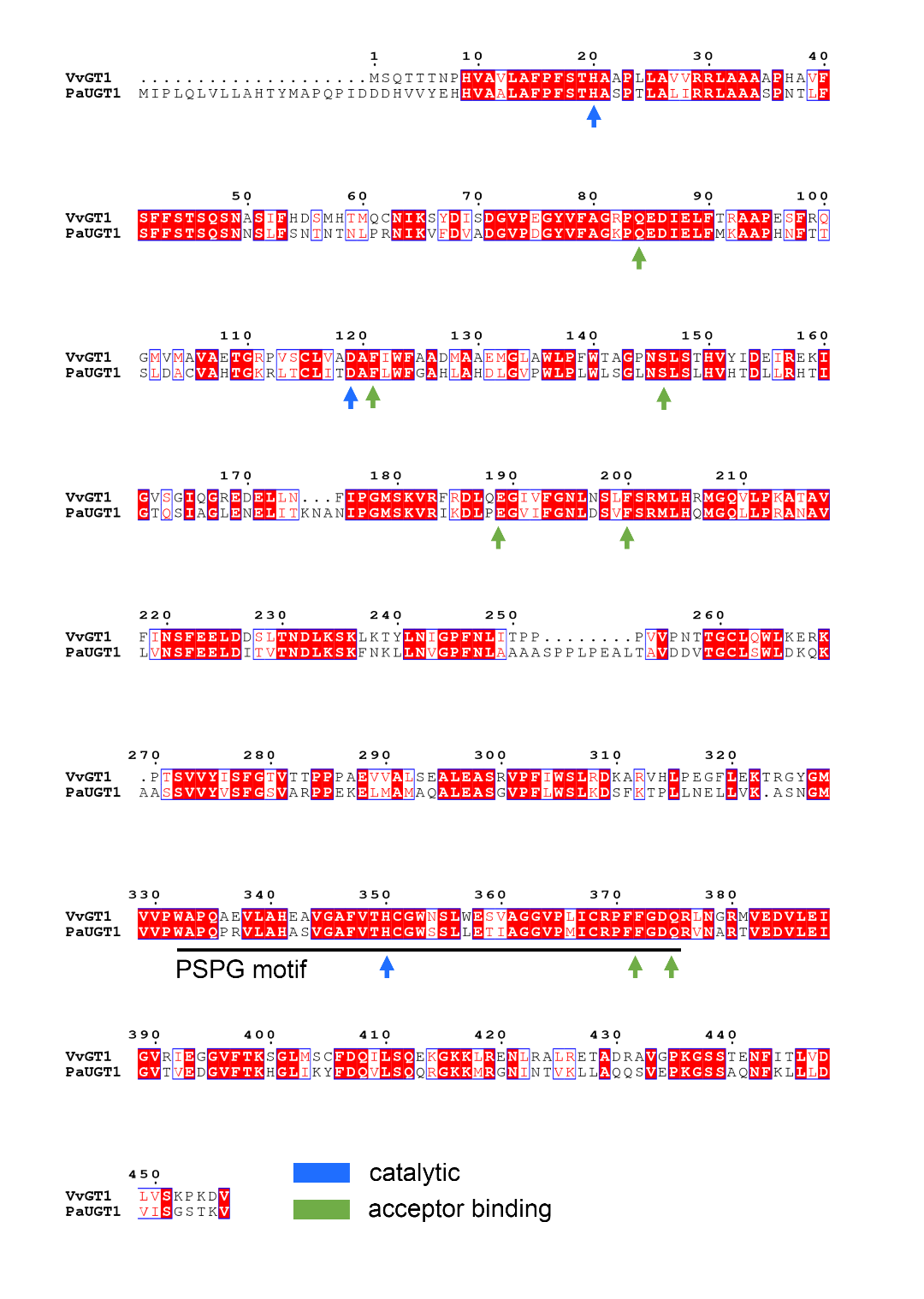


**Figure S2. Sequence alignment of *Vv*GT1 and *Pa*UGT1.**


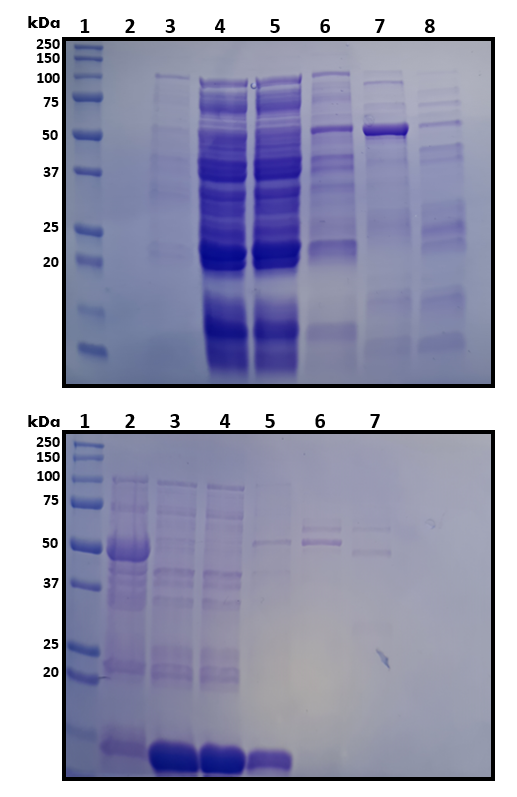


**Figure S3.** Approximate size and purity of recombinant *Pa*UGT1 and *Pa*UGT2 proteins. **A)** SDS-PAGE analysis of recombinant *Pa*UGT1 during purification (Coomassie blue staining). Lane 1: pre-stained denatured protein marker, 2: blank, 3: pellet, 4: supernatant, 5: flowthrough, 6: wash, 7-8: elution fractions, with lane 7 showing 250 mM imidazole elution fraction used for assays. **B)** SDS-PAGE analysis of recombinant *Pa*UGT2 during purification. Lane 1: pre-stained denatured protein marker, 2: pellet, 3: supernatant, 4: flowthrough, 5: wash, 6-7: elution fractions, with lane 6 showing 250 mM imidazole elution fraction used for assays.


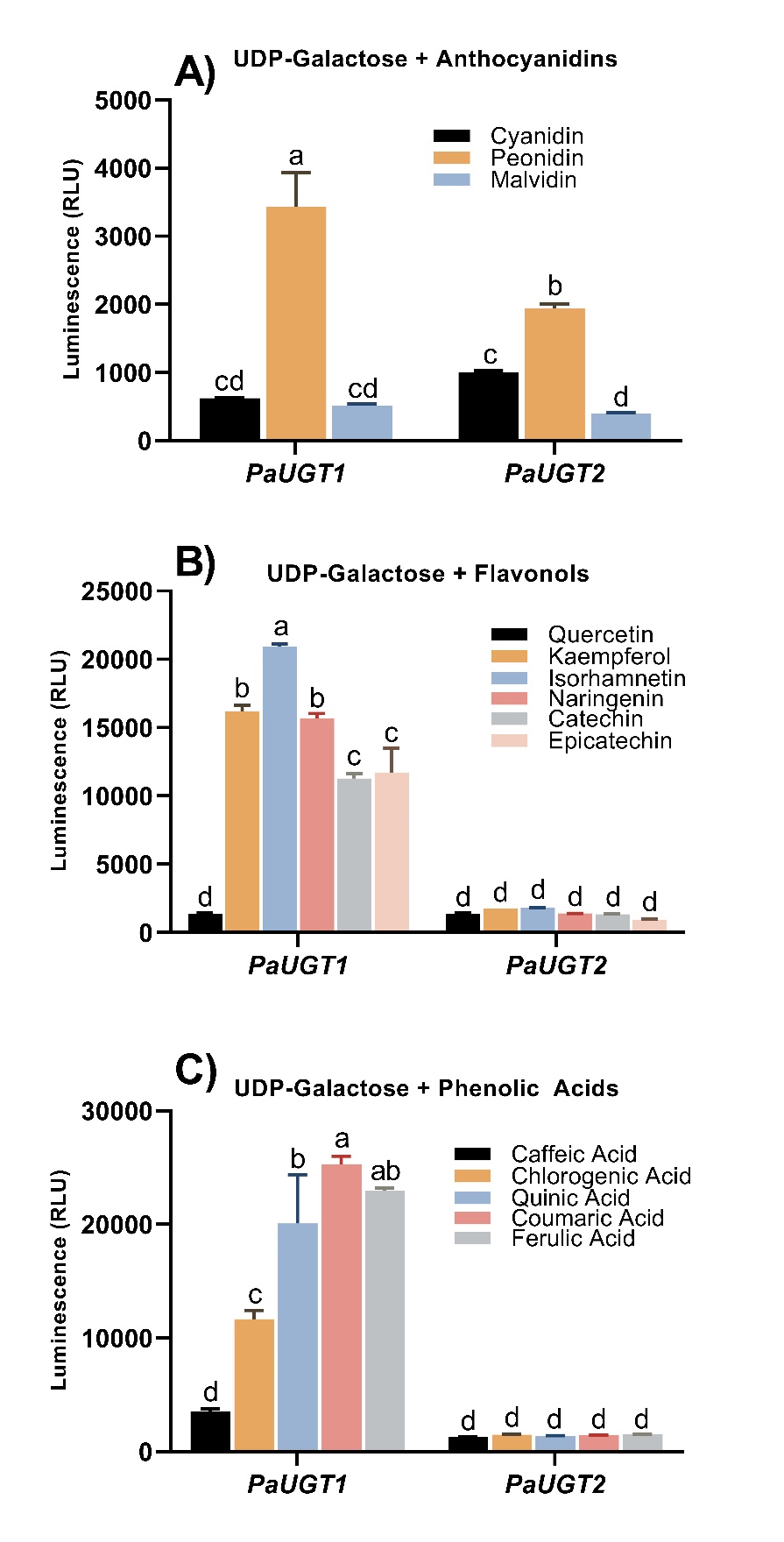


**Figure S4.** *Pa*UGT1 and *Pa*UGT2 recombinant proteins reacted with UDP-Gal and a range of sugar acceptor phenolic compounds to determine substrate specificity. The level of activity was determined by quantifying the amount of UDP released during the reaction, which was measured using the coupled UDP-Glo Glycosyltransferase Assay Kit. A) UDP-Gal and anthocyanidins, B) UDP-Gal and flavonols, C) UDP-Gal and phenolic acids.


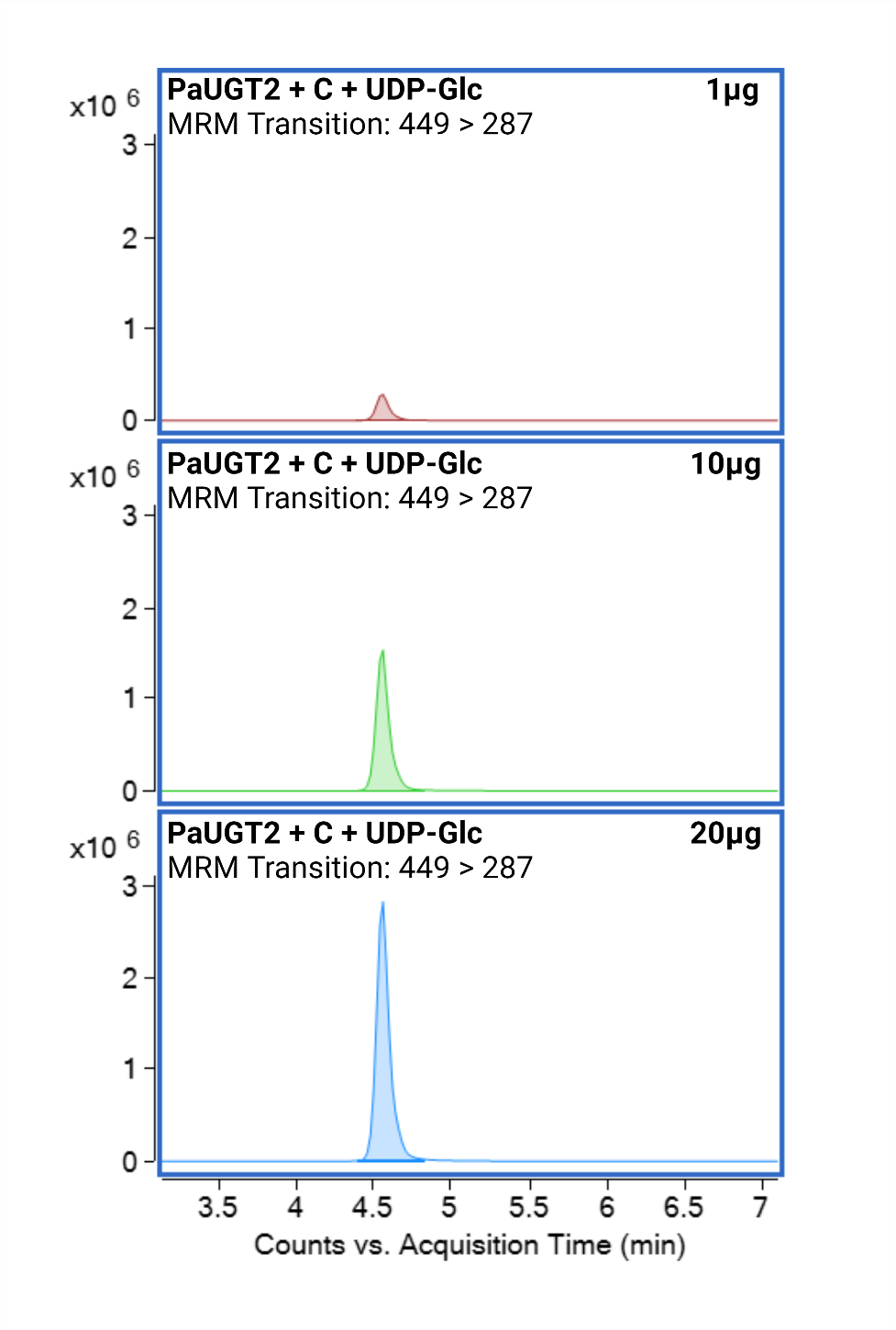


**Figure S5.** LCMS-QQQ data demonstrating the catalytic activity of *Pa*UGT2 with cyanidin (C) and UDP-Glc as substrates. Reaction conditions were as follows: 100 µL total volume containing 500 µM sugar acceptor, 1 mM sugar donor in 50 mM tris-glycine buffer pH 8.0. All reactions were left for 1 h to incubate at room temperature with either 1, 10 or 20 µg of *Pa*UGT1 protein added, and an equal volume of 100% MeOH was added to terminate the reaction. All reaction products were analyzed on an LCMS-QQQ instrument in positive ionisation and multiple reaction monitoring (MRM) mode.


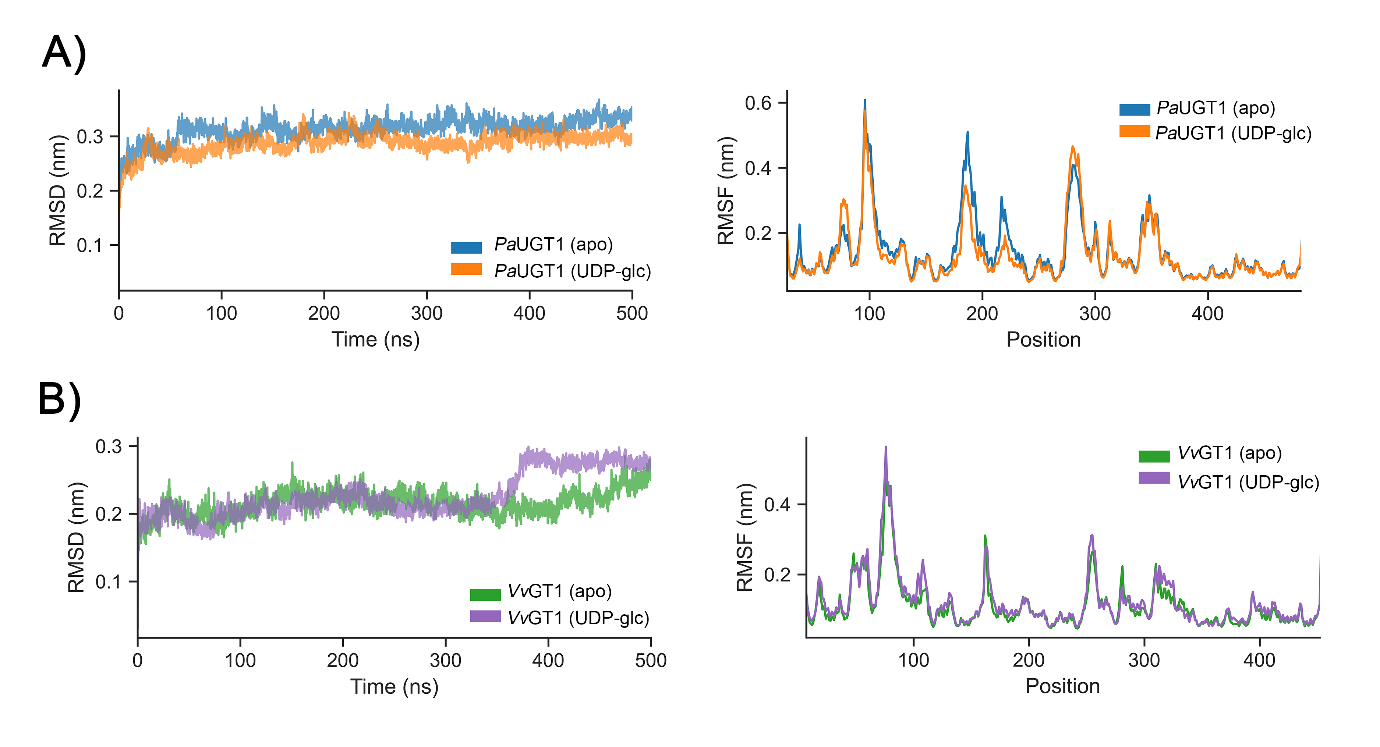


**Figure S6. Structural modelling of *Pa*UGT1 and *Vv*GT1. A)** (left) Average root-mean square deviation (RMSD) of protein backbone atoms for *Pa*UGT1 with and without (apo) UDP-Glc; (right) average root-mean square fluctuation (RMSF) per residue (C-α atoms) with and without (apo) UDP-Glc. **B)** As above, except for *Vv*GT1. In both A) and B) data were averaged over three independent 500 ns molecular dynamics simulations.
